# Cost-effectiveness of a population-based AAA screening program for men over 65 years old in Iran

**DOI:** 10.1186/s12962-021-00283-7

**Published:** 2021-05-13

**Authors:** Rajabali Daroudi, Omid Shafe, Jamal Moosavi, Javad Salimi, Yahya Bayazidi, Mohammad Reza Zafarghandi, Majid Maleki, Majid Moini, Pezhman Farshidmehr, Parham Sadeghipour

**Affiliations:** 1 Department of Health Economics and Management, School of Public Health, Tehran University of Medical Sciences, Tehran, Iran; 2 Cardiovascular Intervention Research Center, Rajaie Cardiovascular Medical and Research Center, Iran University of Medical Sciences, Vali-Asr Ave, 1995614331 Tehran, Iran; 3 Vascular and Endovascular Department, Sina Hospital, Tehran University of Medical Science, Tehran, Iran; 4 Department of Pharmacoeconomics and Pharmaceutical Administration, School of Pharmacy, Tehran University of Medical Sciences, Tehran, Iran

**Keywords:** Abdominal aortic aneurysm, Cost-effectiveness, Screening, Open surgical repair, Endovascular aneurysm repair

## Abstract

**Background:**

Screening program tend to recognized patients in their early stage and consequently improve health outcomes. Cost-effectiveness of the abdominal aortic aneurysm (AAA) screening program has been scarcely studied in developing countries. We sought to evaluate the cost-effectiveness of a screening program for the abdominal aortic aneurysm (AAA) in men aged over 65 years in Iran.

**Methods:**

A Markov cohort model with 11 mutually exclusive health statuses was used to evaluate the cost-effectiveness of a population-based AAA screening program compared with a no-screening strategy. Transitions between the health statuses were simulated by using 3-month cycles. Data for disease transition probabilities and quality of life outcomes were obtained from published literature, and costs were calculated based on the price of medical services in Iran and the examination of the patients’ medical records. The outcomes were life-years gained, the quality-adjusted life-year (QALY), costs, and the incremental cost-effectiveness ratio (ICER). The analysis was conducted for a lifetime horizon from the payer’s perspective. Costs and effects were discounted at an annual rate of 3%. Uncertainty surrounding the model inputs was tested with deterministic and probabilistic sensitivity analyses.

**Results:**

The mean incremental cost of the AAA screening strategy compared with the no-screening strategy was $140 and the mean incremental QALY gain was 0.025 QALY, resulting in an ICER of $5566 ($14,656 PPP) per QALY gained. At a willingness-to-pay of 1 gross domestic product (GDP) per capita ($5628) per QALY gained, the probability of the cost-effectiveness of AAA screening was about 50%. However, at a willingness-to-pay of twice the GDP per capita per QALY gained, there was about a 95% probability for the AAA screening program to be cost-effective in Iran.

**Conclusions:**

The results of this study showed that at a willingness-to-pay of 1 GDP per capita per QALY gained, a 1-time AAA screening program for men aged over 65 years could not be cost-effective. Nevertheless, at a willingness-to-pay of twice the GDP per capita per QALY gained, the AAA screening program could be cost-effective in Iran. Further, AAA screening in high-risk groups could be cost-effective at a willingness-to-pay of 1 GDP per capita per QALY gained.

## Background

An aneurysm is characterized as a pathological, permanent dilation of a vessel [1]. The main agreed-upon definition of the problem is based on the diameter size of the vessel. For an abdominal aortic aneurysm (AAA), a diameter size of greater than 3 cm, with an increased risk for rupture, is accepted as an aneurysm [2].

Investigations in the United States, United Kingdom, Sweden, and Australia have indicated that AAA may affect 1.6% to 7.2% of the population older than 50 years. Also, in terms of prevalence, AAA is 4 to 15 times more common in men than in women and its incidence rises in tandem with age [3–6].

Approximately 200,000 cases with AAA are diagnosed annually in the United States, with nearly 15,000 cases of this total at high risk of rupture. The aortic rupture is often fatal, and between 59 and 83% of these patients expire before admission to the hospital. After acute rupture, the mortality rate of emergent surgery is more than 40%. Indeed, only between 10 and 25% of cases with aortic rupture are likely to survive until discharge time [7–9].

The risk of rupture is related to the size of the aneurysm. In other words, an increase in the size of the dilation is concomitant with an increase in the chance of rupture. The risk of aortic rupture in women is 4 times that in men and twice as high in smokers as in nonsmokers [10, 11].

There is currently a dearth of data on the global economic burden and total cost of AAA; however, the average hospitalization time and the cumulative cost of unruptured and ruptured aneurysms are, respectively, 6.7 and 10.7 days and $59,000 and $93,000 [12].

Open surgical repair (OSR) and endovascular aneurysm repair (EVAR) are 2 accepted treatment modalities for AAA. Research shows that the short-term morbidity rate in EVAR is lower than that in OSR; still, it appears that EVAR increases the risk of reoperation and long-term mortality [13–16]. Such findings denote the usefulness of screening methods for the early diagnosis of AAA, especially in light of the fact that not only are most patients with AAA asymptomatic and complications increase the mortality rate but also therapeutic interventions are expensive. The most common screening strategies for the early detection of AAA are ultrasound sonography and computed tomography angiography (CTA). Aortic sizing is reportedly more accurate on CTA, at the expense of radiation exposure and the risk of contrast-induced kidney injury. Ultrasound sonography is now deemed the gold standard for AAA screening on the strength of its accessibility, generalizability, and safety [17–19].

In the early years of the current century, several randomized clinical trials showed that ultrasound screening for AAA had a significant effect on reducing its mortality [20–22]. Similarly, several economic evaluation studies in Western countries have reported that AAA screening in men over 65 years old is highly cost-effective [23–27]. Such results have prompted some medical societies such as the European Society for Vascular Surgery and the United States Preventive Services Taskforce to recommend AAA screening [28, 29], paving the way for the implementation of nationwide AAA screening programs in several Western countries [30].

The Healthcare system in Iran comprises three sectors including public, private and not-for-profit. Although all sectors are involved in providing secondary and tertiary healthcare services, Primary healthcare services such as maternal and child care, screening programs, etc., are mainly funded and provided by the public sector [31]. National Health Network was one of the major health reforms in Iran intended to decrease the inequities and expand the health care coverage in deprived areas. The addition of family physician program also increases the potentiality for a more homogenous coverage of health facilities [32]. Screening as a preventive community-based intervention play a pivotal role in a primary health care system. Several national screening program mainly on cancer prevention have been launched, however, most of them are not based on national studies and lack cost–benefit analysis [31]. The problem is more aggravated in cardiovascular diseases like aortic aneurysm. Research in Asia is hampered by the current paucity of information about the prevalence of AAA. Be that as it may, the available data indicate that the prevalence of the disease in Asian countries is lower than that in European and American countries [33–35]. Accordingly, given the uncertainty as to the cost-effectiveness of AAA screening in Asian countries, we aimed to evaluate the cost-effectiveness of a 1-time screening program for AAA in men aged over 65 years in Iran.

## Methods

### Model structure

**Fig. 1:**
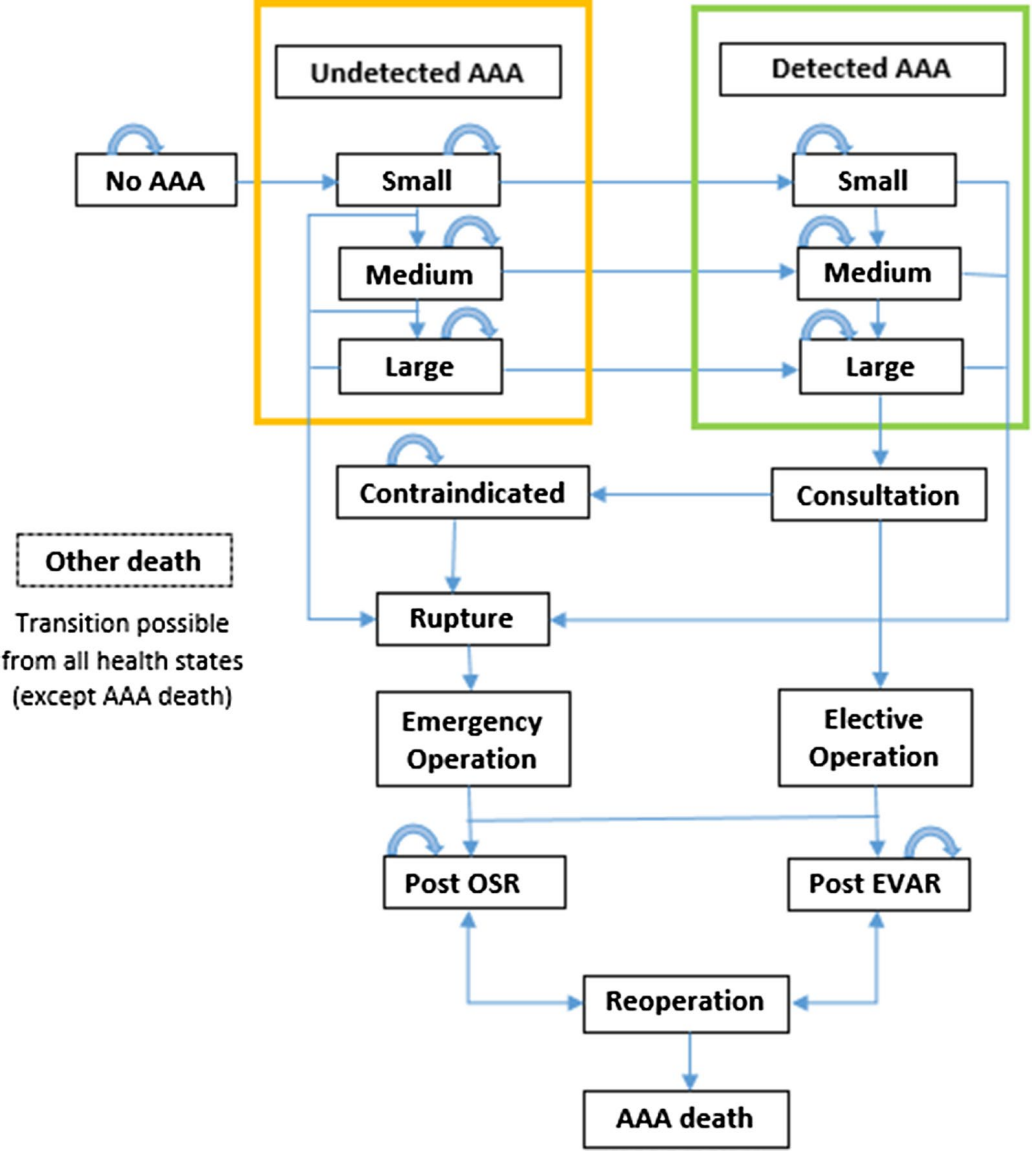
Markov model for both the screening strategy and the no-screening strategy. *AAA* abdominal aortic aneurysm, *EVAR* endovascular aneurysm repair, *OSR* open surgical repair

In the present study, a previously published Markov model [36, 37] was employed to evaluate the long-term costs and clinical outcomes of the screening strategy in comparison with the no-screening strategy (Fig. 1). The Markov model considers 11 mutually exclusive health statuses to simulate AAA progression. The health statuses in the model are as follows: no AAA, 6 AAA statuses (3 undetected AAAs and 3 detected AAAs), 2 postoperative statuses (post-EVAR and post-OSR), death from AAA, and death from other causes. Each of the 3 AAA statuses represents different sizes of aneurysms: small (3–4.4 cm), medium (4.4–5.4 cm), and large (> 5.5 cm). Each arrow in Fig. 1 characterizes a possible transition. Undetected AAAs may be detected by screening or opportunistically. If an aneurysm is detected, ultrasound monitoring is conducted annually for small AAAs and every 6 months for medium AAAs. Patients with aneurysms greater than 5.5 cm undergo elective repair surgery. Rupture may occur in all detected and undetected AAAs. Rupture is followed by 2 scenarios: either patients may die and transit to the status of death from AAA or they may undergo an emergency repair surgery. OSR and EVAR are the suggested surgical interventions for both elective and emergency repair operations. The main complications of surgical methods are myocardial infarction, stroke, renal failure, and death. After surgery, patients may transit to one of the 2 postoperative statuses depending on the type of surgery or die and transit to the status of death from AAA. In all living statuses, patients may die due to other causes and transit to the status of death from other causes.

### Model parameters

The parameters of the Markov model utilized in the current investigation are depicted in Table 1. The prevalence of AAA was extracted from a previous study in Iran and studies in other countries [38–40]. On the assumption that the size distribution of aneurysms in Iran was similar to that in the United Kingdom, the value at the initial cycle was obtained from an AAA screening program run by that country’s National Health System [36]. The probabilities of transition between the AAA statuses and the probabilities of rupture in the different AAA statuses were extracted from a systematic review study [41] and an economic evaluation study conducted in the United Kingdom [36]. The proportion of each surgical type in Iran was obtained by an expert panel. Further, the probability of death after emergency and elective surgery was extracted from previous studies conducted in Iran and other countries [36, 37, 42–44]. The probabilities of reoperation after EVAR and OSR and health-related quality of life for men of different ages were extracted from a study by Zarrouk et al. [37]. Disutility due to surgery was obtained from an investigation by Lederle et al. [45]. Age-specific all-cause mortality rates for the Iranian population were obtained from the available Iranian life tables [46].

**Table 1:** Parameters used in the Markov model to evaluate screening for AAA

Variable	Point estimate (range)	Distribution	Source
Prevalence of AAA	0.03 (0.01–0.04)	Beta	[36–38]
Proportion of small AAAs	0.789	Dirichlet	[34]
Proportion of medium AAAs	0.12	Dirichlet	[34]
Proportion of large AAAs	0.091	Dirichlet	[34]
Transition probabilities (3-monthly)			
From no AAA to small AAA	0.00207 (0.0013–0.0029)	Gamma	[34]
From small AAA to medium AAA	0.037 (0.033–0.042)	Beta	[39]
From medium AAA to large AAA	0.175 (0.119–0.231)	Beta	[39]
Probability of rupture (3-monthly)			
In small AAA	0.00023 (0.00013–0.00038)	Beta	[39]
In medium AAA	0.00160 (0.00043–0.00588)	Beta	[39]
In undetected large AAA	0.0282 (0.01974–0.03666)	Beta	[34]
In detected large AAA	0.0125 (0.008–0.018)	Beta	[34]
In detected large AAA contraindicated for surgery	0.0282 (0.01974–0.03666)	Beta	[34]
Probability of opportunistic detection	0.0114 (0.00798–0.01482)	Beta	[34]
Probability of emergency surgery after rupture	0.368 (0.200–0.500)	Beta	[34]
Probability of elective surgery if large AAA	0.918 (0.85–0.95)	Beta	[35]
Probability of reoperation after EVAR (year 1–2)	0.063 (0.0441–0.0819)	Beta	[35]
Probability of reoperation after OSR (year 1)	0.039 (0.0273–0.0507)	Beta	[35]
Proportion of patients undergoing screening (%)	0.75 (0.65–0.85)	Beta	Expert opinion
Proportion of EVAR as emergency surgery (%)	0.05 (0–0.1)	Beta	Expert opinion
Proportion of EVAR as elective surgery (%)	0.8 (0.7–0.9)	Beta	Expert opinion
Death after^a^			
Elective EVAR	0.013 (0.004–0.023)	Beta	[35, 40, 42]
Elective OSR	0.030 (0.021–0.050)	Beta	[34, 42]
Emergency EVAR	0.307 (0.152–0.492)	Beta	[35, 42]
Emergency OSR	0.5 (0.35–0.65)	Beta	[41]
Health-related quality of life (EQ-5D index)			
65–69 years old	0.83	NA	[35]
70–74 years old	0.81	NA	[35]
75–79 years old	0.79	NA	[35]
80+ years old	0.74	NA	[35]
Health-related disutilities (EQ-5D index)			
Post EVAR and OSR	− 0.02	NA	[43]
All-cause mortality	Age-specific	NA	[44]
Discount rate (%)	3 (0–6)	NA	Expert opinion
Cost of			
EVAR (per patient) (US$)	12,433 (8289–20,722)	Gamma	Our estimation
OSR (per patient) (US$)	6442 (4295–10,737)	Gamma	Our estimation
Screening test (ultrasound) per patient (US$)	39.17 (25.25–57.29)	Normal	Our estimation
Small AAA follow-up (per cycle) (US$)	9.79 (6.31–14.32)	Normal	Our estimation
Medium AAA follow-up (per cycle) (US$)	19.58 (12.63–28.64)	Normal	Our estimation

*NA* not applicable, *AAA* abdominal aortic aneurysm, *EVAR* endovascular aneurysm repair, *OSR* open surgical repair

^a^Mortality rates related to AAA repair were defined as those that occurred within 30 days after surgery

The cost for the screening and follow-up of a patient with a detected small or large AAA was calculated based on the price of physician visits and ultrasound examinations in Iran in 2017. The costs of EVAR and OSR were estimated by examining the medical records of 110 hospitalized patients in 2 referral hospitals in Iran (Rajaie Cardiovascular Medical and Research Center and Sina Hospital). Only direct medical costs were calculated; these costs encompassed those of all diagnostic and treatment modalities—namely hospitalization, laboratory, imaging, physician visit, surgery, complications, and treatment. All costs were converted to US dollars according to the official exchange rate, which was $1 = 34,212 IR Rials [the purchasing power parity (PPP) exchange rate $1 = 12,993 IR Rials] in 2017 [47].

### Analysis

Two identical cohorts of men aged 65 years old were simulated. The first cohort was invited to be screened with ultrasound sonography, but the second cohort was not invited. The model applied a 3-month cycle length for a lifetime horizon. All future costs and effects were discounted at a 3% annual rate. A half-cycle correction was used for both costs and effects on the assumption that both costs and effects occur halfway through a model cycle. The outcomes were life-years gained, the quality-adjusted life-year (QALY), and costs. The analysis was carried out for a lifetime horizon from the point of view of the payer. Additionally, the incremental cost-effectiveness ratio (ICER) was calculated to compare the 2 strategies of screening and no screening. The TreeAge Pro software (TreeAge Pro Software, Inc, Williamston, MA) was utilized for data modeling.

### Sensitivity analysis

Deterministic and probabilistic sensitivity analyses were undertaken to allow for parameter uncertainty. The range used for the uncertainty around the point estimate of each variable and the distribution used in the probabilistic sensitivity analysis are presented in Table 1.

## Results

### Base-case analysis

The main results from the base-case analysis are shown in Table 2. The mean incremental cost of the AAA screening strategy compared with the no-screening strategy was $140 ($369 PPP) and the mean incremental QALY gain was 0.025 QALY, resulting in an ICER of $5566 ($14,656 PPP) per QALY gained. The incremental health gain in terms of life-year was 0.034, yielding an ICER of $4106 ($10,812 PPP) per life-year gained.

**Table 2:** Incremental cost-effectiveness ratio of an abdominal aortic aneurysm screening strategy compared with no screening

Strategy	Average			Incremental			ICER	
	Cost (US$)	QALY	LYG	Cost (US$)	QALY	LYG	Cost (US$) per QALY	Cost (US$) per LYG
No screening	267	8.780	10.890	–	–	–	–	–
Screening	407	8.806	10.924	140	0.025	0.034	5566	4106

*QALY* quality-adjusted life-year, *LYG* life-years gained, *ICER* incremental cost-effectiveness ratio

### Sensitivity analysis

**Fig. 2:**
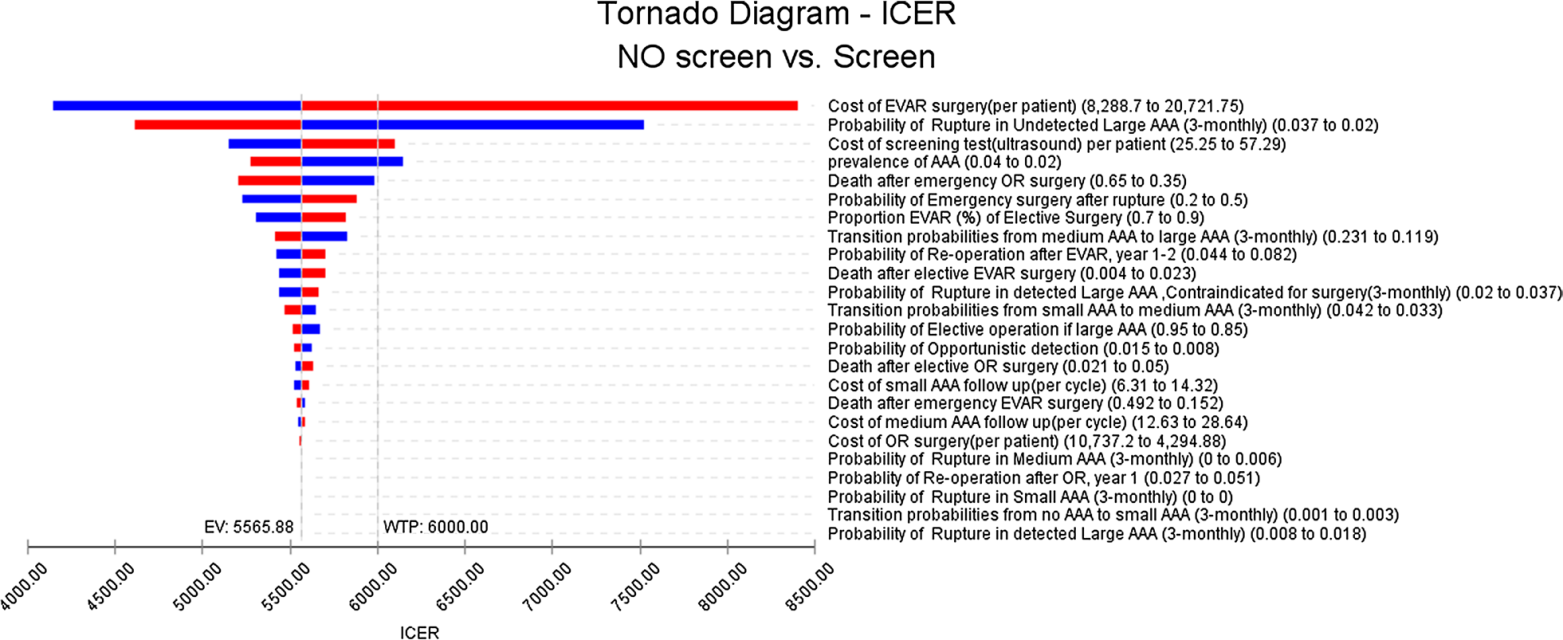
Tornado diagram for 1-way sensitivity analyses

Most of the parameters in the 1-way sensitivity analysis had a limited effect on the results. The variables that influenced the model outcomes the most were the discount rate, the cost of EVAR, the probability of rupture in undetected large AAAs, the cost of screening tests, and the prevalence of AAA (Fig. 2). The results of the 1-way sensitivity analysis for the selected variables are presented in Table 3. At a 6% discount rate on health gains and costs, the ICER changed to about $7334 per QALY gained. (The undiscounted equivalent was about $4151 per QALY gained). When the cost of EVAR was increased to $20,722, the ICER rose to $8409 per QALY gained, while decreasing the cost of EVAR to $8289 diminished the ICER to $4144 per QALY gained. A change in the probability of the rupture of undetected large AAAs between the lower and upper limits led to a variation of the ICER of between $7524 and $4618 per QALY gained. Moreover, an increase or a decrease in the cost of screening tests by about 40% resulted in a variation of the ICER of between $5153 and $6103 per QALY gained. Finally, when the prevalence of AAA was reduced to 1%, the ICER rose to $7890 per QALY gained; and when the prevalence was increased to 4%, the ICER dropped to $5275 per QALY gained.

**Table 3:** Result of the 1-way sensitivity analysis of the selected variables

Variable	Cost (s)			QALY			ICER ($ per QALY)
	No screening	Screening	Increment	No screening	Screening	Increment	
Discount rate (%)							
0	364	512	148	11.005	11.040	0.036	4151
0.6	199	332	133	7.182	7.200	0.018	7334
Cost of EVAR ($)							
8289	203	308	104	8.780	8.806	0.025	4144
20,722	393	604	212	8.780	8.806	0.025	8409
Probability of the rupture in undetected large AAAs (3-monthly)							
0.0197	267	406	139	8.799	8.817	0.018	7524
0.0367	266	406	141	8.765	8.796	0.031	4618
Cost of the screening test (ultrasound) per patient ($)							
25	267	396	130	8.780	8.806	0.025	5153
57	267	420	154	8.780	8.806	0.025	6103
Prevalence of AAA (%)							
0.01	235	338	103	8.795	8.812	0.017	7890
0.04	298	475	177	8.766	8.799	0.034	5275
Probability of death after emergency OSR							
0.35	267	407	140	8.786	8.809	0.023	5984
0.65	266	406	140	8.775	8.802	0.027	5203
Probability of emergency surgery after rupture							
0.2	241	387	147	8.771	8.800	0.028	5233
0.5	287	422	135	8.787	8.810	0.023	5886
Proportion of EVAR as elective surgery (%)							
0.7	255	388	133	8.780	8.805	0.025	5311
0.9	278	425	147	8.781	8.806	0.025	5818
Transition probabilities from medium AAA to large AAA (3-monthly)							
0.119	257	391	135	8.788	8.811	0.023	5833
0.231	271	414	143	8.776	8.802	0.026	5419
Probability of death after elective EVAR							
0.004	267	407	140	8.781	8.807	0.026	5443
0.023	266	406	140	8.780	8.804	0.025	5709

*AAA* abdominal aortic aneurysm, *EVAR* endovascular aneurysm repair, *OSR* open surgical repair

### Probabilistic sensitivity analysis

**Fig. 3:**
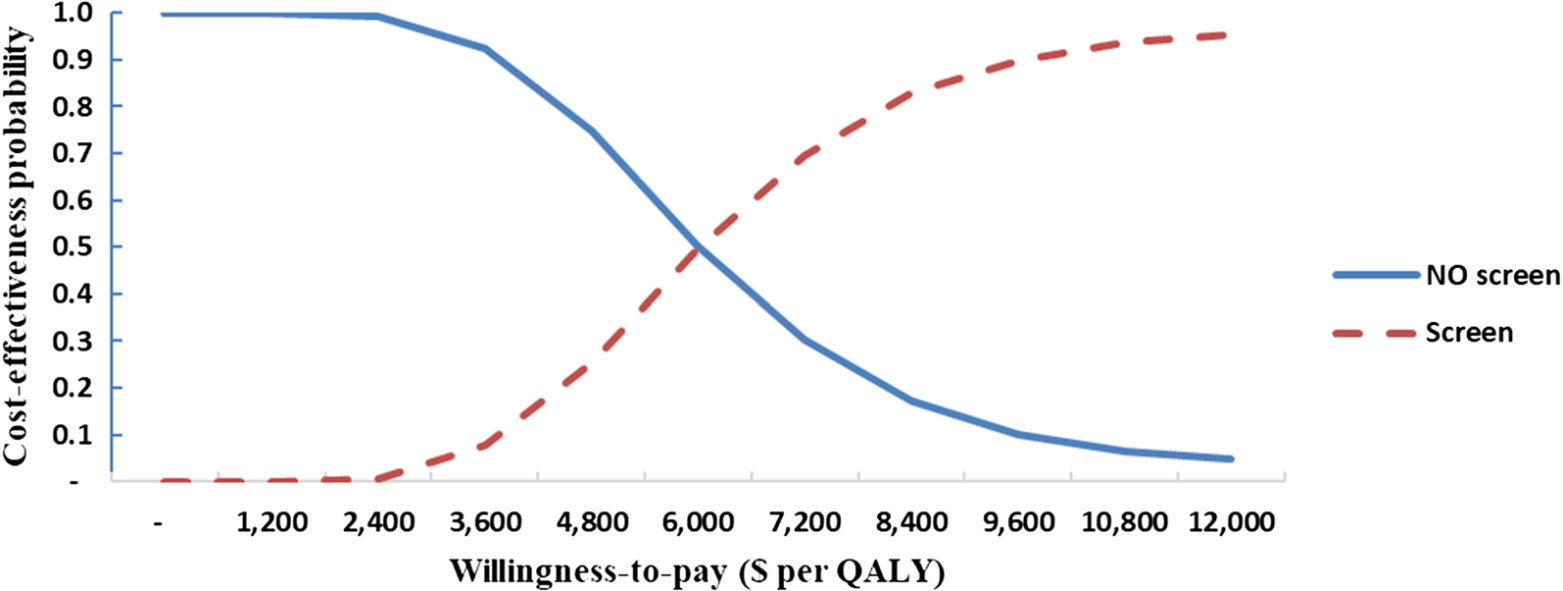
Cost-effectiveness acceptability curve for abdominal aortic aneurysm screening. *QALY* quality-adjusted life-year

The cost-effectiveness acceptability curve at different thresholds of willingness to pay (Fig. 3) shows that the probability of the cost-effectiveness of AAA screening was 0.5 at a threshold of $6000 per QALY gained and 0.95 at a threshold of $12,000 per QALY gained.

## Discussion

In this study, we investigated the cost-effectiveness of a 1-time AAA screening program for men aged over 65 years in Iran. Our analysis showed that at a willingness-to-pay of 1 gross domestic product (GDP) per capita ($5628 based on real market exchange rate and $14,819 based on PPP exchange rate) per QALY gained, the probability of the cost-effectiveness of AAA screening was about 50%; while at a willingness-to-pay of twice the GDP per capita per QALY gained, there was approximately a 95% probability for the AAA screening program to be cost-effective in Iran. To the best of our knowledge, the present study is the only investigation to analyze the cost-effectiveness of an AAA screening program in a developing country.

Our sensitivity analysis revealed that our results were sensitive to the prevalence of AAA. Based on our 1-way sensitivity analysis results, AAA screening could be cost-effective at a threshold of 1 GDP per capita ($5628) per QALY gained, if the prevalence of AAA in Iran was 4% or greater. Furthermore, AAA screening could be cost-effective at a threshold of twice the GDP per capita per QALY gained, if the prevalence of AAA in Iran was 1% or greater.

Recent years have witnessed a change in the epidemiology of AAA in Western countries, the most striking of which is the significantly reduced prevalence rate of AAA owing to alterations in smoking habits, followed by the improved management of cardiovascular risk factors [35, 48]. Although these epidemiological changes have raised questions over the cost-effectiveness of AAA screening, the results of recent economic evaluation studies in Western countries have demonstrated that AAA screening in men aged 65 years or above is still cost-effective in the new context [36, 37, 44, 49, 50]. There is, however, no evidence of a decrease in the prevalence of AAA in Asian countries, including Iran [33, 40]. Our extensive literature search showed only a few studies on the prevalence of AAA in Iran [39, 51, 52]. The salient point as regards these investigations is that their focus on special patient groups greatly lessens the applicability of their results to the general population of Iran. Mirsharifi et al. [39] reported a prevalence rate of 10% for AAA in men older than 65 years from among patients who referred for ultrasound sonography to 3 different centers in Tehran, Iran, in the year 2008. Shirani et al. [52] found a prevalence rate of 3.8% for AAA in men aged over 65 years from among the candidates for coronary artery bypass graft surgery in Tehran Heart Center. Recent reports on the prevalence of AAA in Europe in the last decade have shown a clear decline significantly related to successful strategies for risk factor modification [37, 53]. In contrast, the current status of the strategies aimed at modifying AAA risk factors in developing countries would logically hint at a higher rate of AAA incidence. Assuming a comparable status concerning the prevalence of AAA in the general populations of men aged 65 years or above in Europe and Iran, we can conclude that AAA screening could not be cost-effective. Still, the fact that local studies indicate relatively high rates of AAA in high-risk groups would bolster the argument in favor of screening such patients.

More recently, the results of a systematic review study commissioned by the United States Preventive Services Task Force showed that 1-time AAA screening in men 65 years or older was associated with decreased AAA-related mortality and rupture rates and increased rates of elective surgery, but it was not associated with all-cause mortality benefits and long-term differences in the quality of life resulting from screening [54]. Based on these findings, the said task force recommended 1-time screening for AAA with ultrasonography in men aged 65 to 75 years who have ever smoked and selective screening for AAA with ultrasonography in men aged 65 to 75 years who have never smoked rather than routinely screening all men in this group [55].

The cost of EVAR was another variable that greatly influenced the results of our model. Based on expert opinion in this study, EVAR was more popular as the first therapeutic strategy in patients undergoing AAA treatment in Iran; consequently, we assumed that 80% of elective surgical operations were performed via EVAR. Although by comparison with OSR, the EVAR procedure has a favorable hospital course, its superiority has never been proven and its long-term efficacy has been challenged recently [56, 57]. The result of a systematic review and meta-analysis conducted by Bulder et al. [56] showed that the 30-day mortality rate for EVAR was lower than that with OSR (1.16 vs. 3.27%), but the long-term survival rates were similar for EVAR and OSR (hazard ratio: 1.01, 1.00, and 0.98 for 3, 5, and 10 years, respectively). In another systematic review study, Chen et al. [57] found that OSR and EVAR had similar all-cause mortality over a 5-year follow-up, which was maintained after at least 10 years of follow-up. The authors also reported that a significantly lower proportion of their patients undergoing open repair required re-intervention (odds ratio: 0.38, 95% confidence interval: 0.24 to 0.64), which was maintained over 5 years of follow-up. Based on our estimations, the mean cost of EVAR was about twice the mean cost of OSR in Iran. Therefore, since OSR is comparable with EVAR at least in terms of long-term efficacy, it is possible to boost the likelihood of the cost-effectiveness of AAA screening by using OSR in lieu of EVAR.

## Limitation

In this study, to investigate the cost-effectiveness of AAA screening in Iran, we used a Markov model, the validity of which was confirmed in previous studies [26, 36, 37]. Given the scarcity of data regarding some variables such as the progress and rupture rates of AAA in Iran, in this study, we assumed that the values of these variables in Iran are the same as in western countries. However, these variables may be context-specific and their values in Iran may be different from Western countries. Further research on the natural history of the AAA in Iran is needed to fill this data gap.

## Conclusion

In conclusion, our results showed that at a willingness-to-pay of 1 GDP per capita ($5628) per QALY gained, a 1-time AAA screening program for men aged 65 years or above could not be cost-effective; whereas at a willingness-to-pay of twice the GDP per capita per QALY gained, the AAA screening program could be cost-effective in Iran. In addition, AAA screening in high-risk groups could be cost-effective at a willingness-to-pay of 1 GDP per capita per QALY gained.

## Data Availability

The data and materials of the present study are available.
